# Postoperative pulmonary function of patients with lung cancer and interstitial lung abnormalities

**DOI:** 10.1007/s11748-024-02037-7

**Published:** 2024-05-09

**Authors:** Norifumi Tsubokawa, Takahiro Mimae, Takeshi Mimura, Atsushi Kagimoto, Atsushi Kamigaichi, Nobutaka Kawamoto, Yoshihiro Miyata, Morihito Okada

**Affiliations:** 1 Department of Surgical Oncology, Hiroshima University, 1–2-3, Kasumi, Minami-ku, Hiroshima, 734-8551 Japan; 2 Department of General Thoracic Surgery, National Hospital Organization Kure Medical Center and Chugoku Cancer Center, Kure, Japan

**Keywords:** Interstitial lung abnormality, Lung resection, Pulmonary function

## Abstract

**Objective:**

We investigated the impact of radiological interstitial lung abnormalities on the postoperative pulmonary functions of patients with non-small cell lung cancer.

**Methods:**

A total of 1191 patients with clinical stage IA non-small cell lung cancer who underwent lung resections and pulmonary function tests ≥ 6 months postoperatively were retrospectively reviewed. Postoperative pulmonary function reduction rates were compared between patients with and without interstitial lung abnormalities and according to the radiological interstitial lung abnormality classifications. Surgical procedures were divided into wedge resection, 1–2 segment resection, and 3–5 segment resection groups.

**Results:**

No significant differences in postoperative pulmonary function reduction rates 6 months after wedge resection were observed between the interstitial lung abnormality [n = 202] and non-interstitial lung abnormality groups [n = 989] [vital capacity [VC]: 6.82% vs. 5.00%; forced expiratory volume in 1 s [FEV1]: 7.05% vs. 7.14%]. After anatomical resection, these values were significantly lower in the interstitial lung abnormality group than in the non-interstitial lung abnormality group [VC: 1–2 segments, 12.50% vs. 9.93%; 3–5 segments, 17.42% vs. 14.23%; FEV1: 1–2 segments: 13.36% vs. 10.27%; 3–5 segments: 17.36% vs. 14.39%]. No significant differences in postoperative pulmonary function reduction rates according to the radiological interstitial lung abnormality classifications were observed.

**Conclusions:**

The presence of interstitial lung abnormalities had a minimal effect on postoperative pulmonary functions after wedge resections; however, pulmonary functions significantly worsened after segmentectomy or lobectomy, regardless of the radiological interstitial lung abnormality classification in early-stage non-small cell lung cancer.

**Supplementary Information:**

The online version contains supplementary material available at 10.1007/s11748-024-02037-7.

## Introduction

Interstitial lung abnormalities [ILAs] are defined as those observed on chest computed tomography [CT] images that are associated with interstitial lung disease or pulmonary fibrosis in individuals without a clinical diagnosis [1]. Patients with ILAs often exhibit restrictive lung deficit development, accelerated lung function decline, poorer subjective health and physical function, and increased mortality rates [1–5]. A higher incidence rate of lung cancer has also been reported for patients with ILAs [4–6]. Although surgical resection is the standard treatment for early-stage non-small cell lung cancer [NSCLC], the long-term survival rate of patients with ILAs is poorer than that of those without ILAs [5, 7, 8]. In addition to lung cancer, other causes, such as respiratory failure, are responsible for the death of patients with ILAs and NSCLC [6, 7]. Because postoperative pulmonary function is vital to a patient’s physical condition and prognosis [9], preserving pulmonary function after lung resection is crucial for patients with ILAs. However, little is known about the long-term postoperative pulmonary function of patients with ILAs. Therefore, we investigated the impact of radiological ILA on the postoperative pulmonary functions in patients with early-stage non-small cell lung cancer.

## Patients and methods

The Institutional Review Boards of Hiroshima University [E2022-0125; approved on August 25, 2022] and the National Hospital Organization of Kure Medical Center and Chugoku Cancer Center [2022–28; approved on September 1, 2022] approved this study and waived the requirement for informed consent because of the retrospective nature of the study.

### Study cohort

We included 1318 patients who underwent complete resection for clinical stage IA NSCLC at Hiroshima University and the National Hospital Organization of Kure Medical Center and Chugoku Cancer Center between January 2010 and December 2020. We excluded 127 patients who did not undergo pulmonary function tests for more than 6 months after surgery. Among the 127 patients excluded from this study, 43 patients had ILAs on HRCT (18 UIP patterns, 12 possible UIP patterns, 13 inconsistent with UIP patterns). Nine patients died within 6 months due to acute exacerbations of interstitial pneumonia (n = 4) and other causes of death (n = 5). Among the four patients who died due to acute exacerbations, one underwent a wedge resection, one a segmentectomy, and the other two lobectomies. One patient was lost to follow-up within 6 months. Thirty-three patients did not undergo pulmonary function tests even though they were alive for more than 6 months. The main reason was the lack of these tests being ordered. A total of 1191 patients were included in the study; 202 had ILAs and 989 did not. TNM staging was performed according to the TNM Classification of Lung and Pleural Tumors [8th edition] [10]. An individual database was prospectively maintained at both two institutions.

The surgical approach and procedures were decided according to the tumor status and patient status. Wedge resection or segmentectomy was performed as an optional procedure for peripheral tumors in patients who underwent complete tumor resection. Especially, patients with small size (≤ 3 cm) and peripheral GGO dominant tumors as an intensive indication, or compromised patients with severely low preoperative pulmonary or cardiac functions as a passive indication underwent wedge resections. If the surgical margin is inadequate in sublobar resection, we consider additional resections or convert to segmentectomy or lobectomy. Systematic lymph node dissection of the hilar or mediastinal nodes was performed during segmentectomy and lobectomy, but not during wedge resection. Complete VATS was often performed in Kure Medical Center and Chugoku Cancer Center, while hybrid VATS was performed in Hiroshima University regardless of surgical procedures. We counted two segments for resection in the right middle lobe, three segments in the right upper lobe, four segments each in the left upper and left lower lobes, and five segments in the right lower lobe. Surgical procedures were divided into three groups: wedge resection group, 1–2 segment resection group, and 3–5 segment resection group.

### Classification of ILA

Preoperative high-resolution computed tomography [CT] images were reviewed by the multidisciplinary tumor board, which includes surgical oncologists, medical oncologists, pulmonologists, radiologists, and pathologists to ensure comprehensive screening for ILAs at both two institutions. Radiologically determined ILAs were defined according to the 2011 American Thoracic Society [ATS], European Respiratory Society [ERS], Japanese Respiratory Society [JRS], and Latin American Thoracic Association [LATA] classifications [11]. Based on these classifications, the results were classified as usual interstitial pneumonia [UIP] pattern, possible UIP pattern, or inconsistent with UIP pattern.

### Pulmonary function tests

Pulmonary function tests were performed preoperatively and 6 and 12 months postoperatively. The patients’ vital capacity [VC] and forced expiratory volume in 1 s [FEV1] were analyzed. The rate of reduction in each postoperative pulmonary function was calculated as follows: [each postoperative parameter at each time point—each preoperative parameter]/ [each preoperative parameter]. We divided the patients into four groups according to the reduction rate in VC and FEV1 by 20% intervals: ≤ 0%, > 0–20%, ≥ 20–40%, and ≥ 40%.

### Statistical analysis

The continuous variables were reported as the medians [interquartile ranges] and were compared using the Mann–Whitney U test. The categorical variables were reported as the numbers [percentages] and compared using Fisher’s exact test. Logistic regression models were used to identify the risk factors for decreased postoperative pulmonary function. A backward stepwise method was used to select variables for the multivariable analysis. All data were analysed using JMP [version 16.0; SAS Institute, Cary, NC, USA]. Statistical significance was indicated by P-values < 0.05.

## Results

### Comparison of postoperative pulmonary function among the ILA and non-ILA groups

**Fig. 1:**
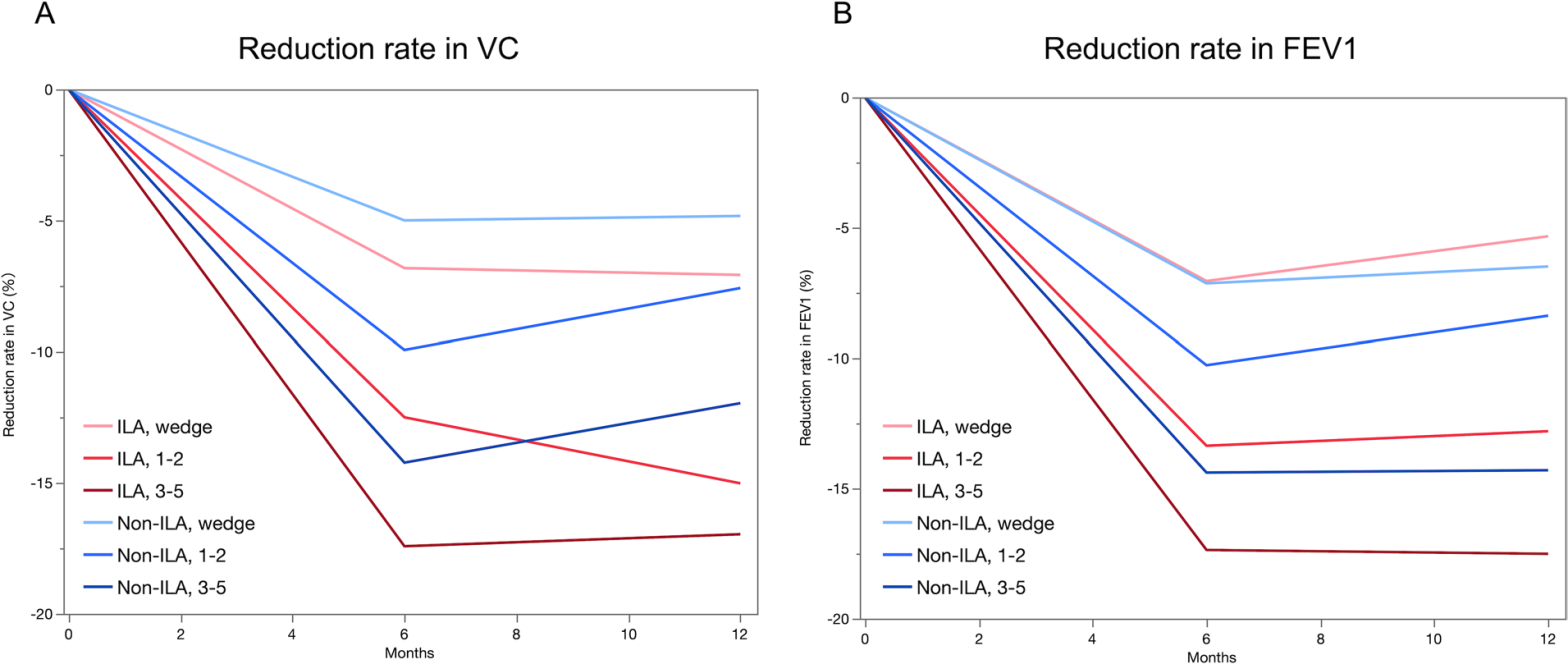
Changes in the postoperative reduction rates of VC [**A**] and FEV1 [**B**] of the ILA and non-ILA groups at 6 and 12 months after surgery. The light red line, red line, and dark red line represent patients with ILAs who underwent wedge resection, 1–2 segment resection, and 3–5 segment resection, respectively. The light blue line, blue line, and dark blue line represent non-ILA patients who underwent wedge resection, 1–2 segment resection, and 3–5 segment resection, respectively. FEV1, forced expiratory volume in 1 s; ILAs, interstitial lung abnormalities; VC, vital capacity

Among the 1191 patients, 202 exhibited ILAs on high-resolution CT images. The characteristics of the patients in the ILA and non-ILA groups are shown in Table 1. Patients with ILA were significantly more likely to be male, have larger solid tumors, and have lower pulmonary function than those without ILAs. Among the 202 patients, the UIP pattern was observed in 45 patients [22%], the possible UIP pattern was observed in 96 patients [48%], and the inconsistent with UIP pattern was observed in 61 patients [33%]. The postoperative reduction rates of VC and FEV1 of patients with and without ILAs according to the surgical procedures are shown in Fig. 1 and Table S1. Postoperative VC and FEV1 of the ILA group did not increase from 6 to 12 months after surgery compared with the values of the non-ILA group. Moreover, no significant difference in the postoperative reduction rates of VC and FEV1 at 6 months after wedge resection was observed between the ILA and non-ILA groups [VC: 6.82% vs. 5.00%, respectively, P = 0.242; FEV1: 7.05% vs. 7.14%, respectively, P = 0.767]. However, after 1–2 segment resection and 3–5 segment resection, these values were significantly lower in the ILA group than in the non-ILA group [VC: 1–2 segments, 12.50% vs. 9.93%, respectively, P = 0.013; 3–5 segments, 17.42% vs. 14.23%, respectively, P = 0.002; FEV1: 1–2 segments, 13.36% vs. 10.27%, respectively, P = 0.139; 3–5 segments, 17.36% vs. 14.39%, respectively, P = 0.046].

**Table 1:** Patient characteristics

	ILA n = 202	Non-ILA n = 989	P value	UIP or possible UIP pattern n = 141	Inconsistent with UIP pattern n = 61	P value
Age, years	74 [69–79]	70 [64–75]	< 0.001	74 [70–78]	73 [69–80]	0.973
Sex [male]	149 [74%]	518 [52%]	<0 .001	114 [81%]	35 [57%]	< 0.001
Brinkman index	920 [525–1200]	100 [0–900]	< 0.001	1000 [600–1320]	635 [205–1000]	0.002
Ground-glass opacity [ +]	72 [36%]	587 [59%]	<0 .001	43 [31%]	29 [48%]	0.002
Solid tumor size [mm]	17 [13–18]	12 [6–18]	<0 .001	18 [14–22]	16 [11–21]	0.064
SUVmax	3.5 [1.8–6.3]	1.7 [1.0–3.5]	<0 .001	4.4 [2.3–6.6]	2.2 [1.3–4.4]	< 0.001
VC [%]	97.5 [86.3–110.7]	101.7 [81.7–112.2]	0.015	95.9 [85.1–106.9]	100 [89.5–115.7]	0.058
VC ≤ 80%	28 (14%)	80 (8%)	0.014	24 (17%)	4 (7%)	0.036
FEV1/FVC [%]	76.4 [68.7–81.5]	76.2 [70.3–81.2]	0.823	76.1[67.6–81.3]	77.2 [69.9–82.1]	0.458
FEV1/ FVC ≤ 70%	61 (30%)	243 (25%)	0.099	45 (32%)	16 (26%)	0.415
DLco [%]	59.9 [45.6–78.2]	83 [69.3–96.4]	< 0.001	58.8 [44.4–75.0]	68 [45.7–90.5]	0.179
DLco ≤ 60%	69 (50%)	102 (14%)	<0.001	57 (53%)	12 (40%)	0.198
UIP pattern	45 [22%]					
Possible UIP pattern	96 [48%]					
Inconsistent with UIP pattern	61 [30%]					
Surgical procedure			0.004			0.002
Wedge resection	63 [31%]	226 [23%]		54 [38%]	9 [15%]	
Segmentectomy	44 [22%]	318 [32%]		27 [19%]	17 [28%]	
Lobectomy	95 [47%]	445 [45%]		60 [43%]	35 [58%]	
1–2 resected segments	51 [25%]	342 [35%]		30 [21%]	21 [34%]	
3–5 resected segments	88 [44%]	421 [43%]		57 [40%]	31 [51%]	
Histological type			<0.001			0.002
Adenocarcinoma	120 [59%]	867 [88%]		73 [52%]	73 [52%]	
Squamous cell carcinoma	68 [34%]	76 [8%]		58 [41%]	10 [16%]	
Others	14 [7%]	46 [5%]		10 [7%]	4 [7%]	
Pathological uip pattern	74 [36%]			59 [42%]	15 [25%]	0.017

Variables are presented as the number [percentage] or median [interquartile range]. DLco, predicted diffusing capacity for carbon monoxide; *FEV*1 forced expiratory volume in 1 s, *FVC* forced vital capacity, *IL* interstitial lung abnormality, *SUVmax* maximum standard uptake value, *UIP* unusual interstitial pneumonia, *VC* vital capacity

**Fig. 2:**
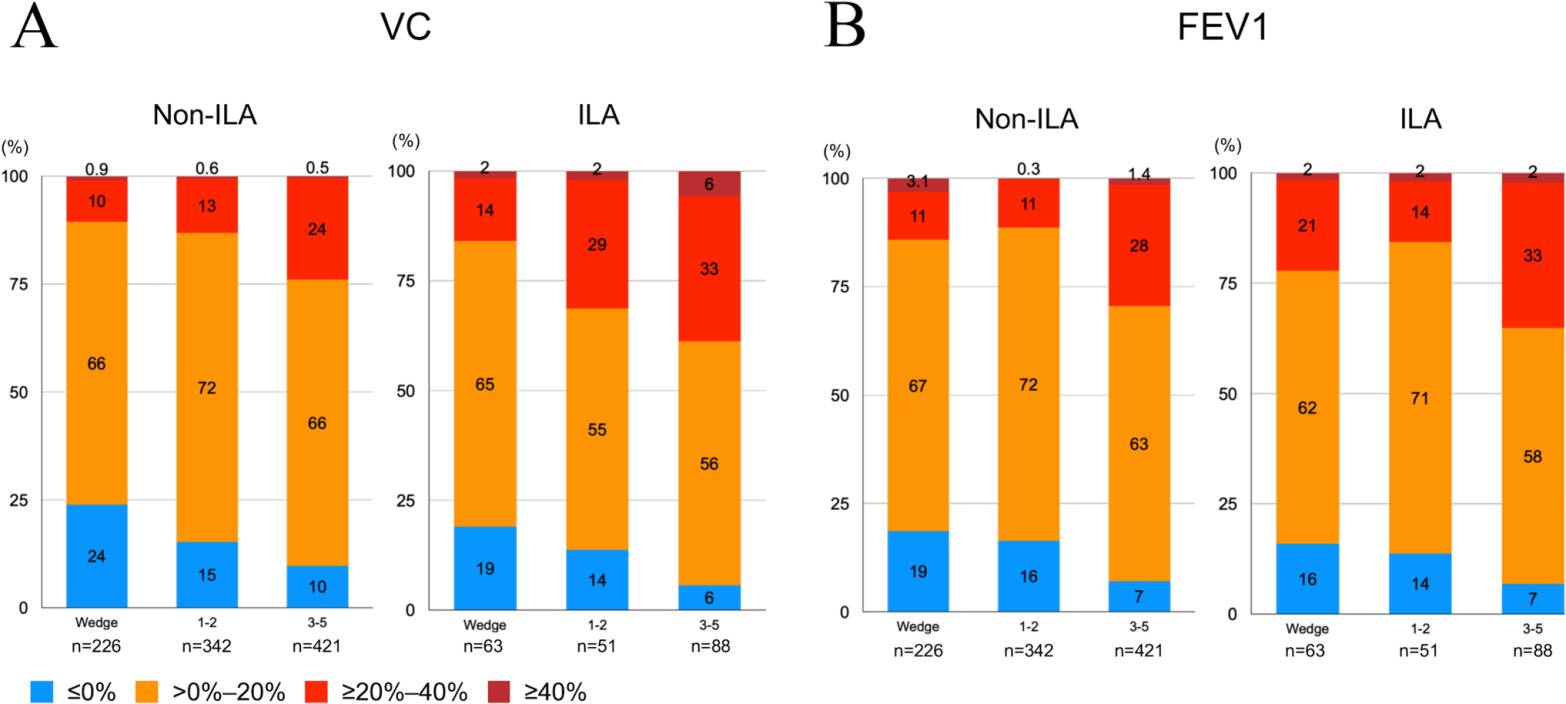
The percentages of patients according to the reduction rate at 6 months after surgery based on 20% intervals for VC [**A**] and FEV1 [**B**] of the ILA and non-ILA groups. FEV1, forced expiratory volume in 1 s; ILA, interstitial lung abnormality; VC, vital capacity

Figure 2 shows the percentages of patients with and without ILAs according to the reduction rate of pulmonary function in 20% intervals at 6 months after surgery. As the number of resected segments increased, the proportion of patients with a reduction rate of ≤ 0% decreased and those with a reduction rate of ≥ 20% increased in both groups. However, the proportions of patients with a reduction rate of ≥ 20%–40% or ≥ 40% were higher in the ILA group than in the non-ILA group for all surgical procedures.

### Comparison of postoperative pulmonary function among radiological interstitial patterns

**Fig. 3:**
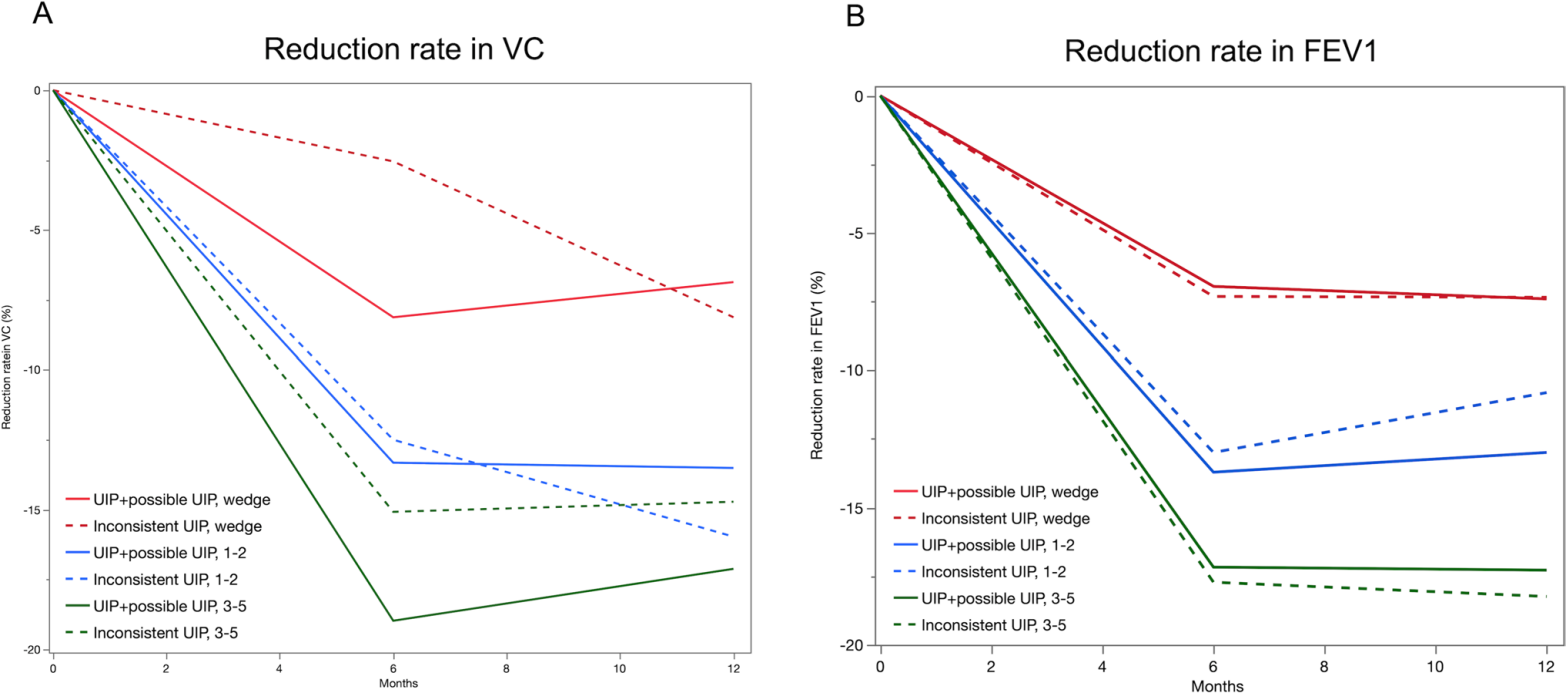
Changes in the postoperative reduction rates of VC [**A**] and FEV1 [**B**] at 6 and 12 months after surgery in the UIP pattern or possible UIP pattern groups and the inconsistent with UIP pattern group. The red line and dotted red line represent patients who underwent wedge resection in the UIP pattern or possible UIP pattern groups and the inconsistent with UIP pattern group, respectively. The blue line and dotted blue line represent patients who underwent 1–2 segment resection in the UIP pattern or possible UIP pattern groups and the inconsistent with UIP pattern group, respectively. The green line and dotted green line represent patients who underwent 1–2 segment resection in the UIP pattern or possible UIP pattern groups and the inconsistent with UIP pattern group, respectively. FEV1, forced expiratory volume in 1 s; UIP, usual interstitial pneumonia; VC, vital capacity

We also compared the postoperative pulmonary function between the UIP pattern or possible UIP pattern [n = 141] and inconsistent with UIP pattern [n = 61]. The patient characteristics of the two groups are shown in Table 1. More male patients and higher Brinkman index were observed in the UIP pattern or possible UIP pattern groups than in the inconsistent with UIP pattern group. No significant difference in the postoperative reduction rates of VC and FEV1 was observed between the two groups according to the surgical procedures performed [Fig. 3 and Table S2].

**Fig. 4:**
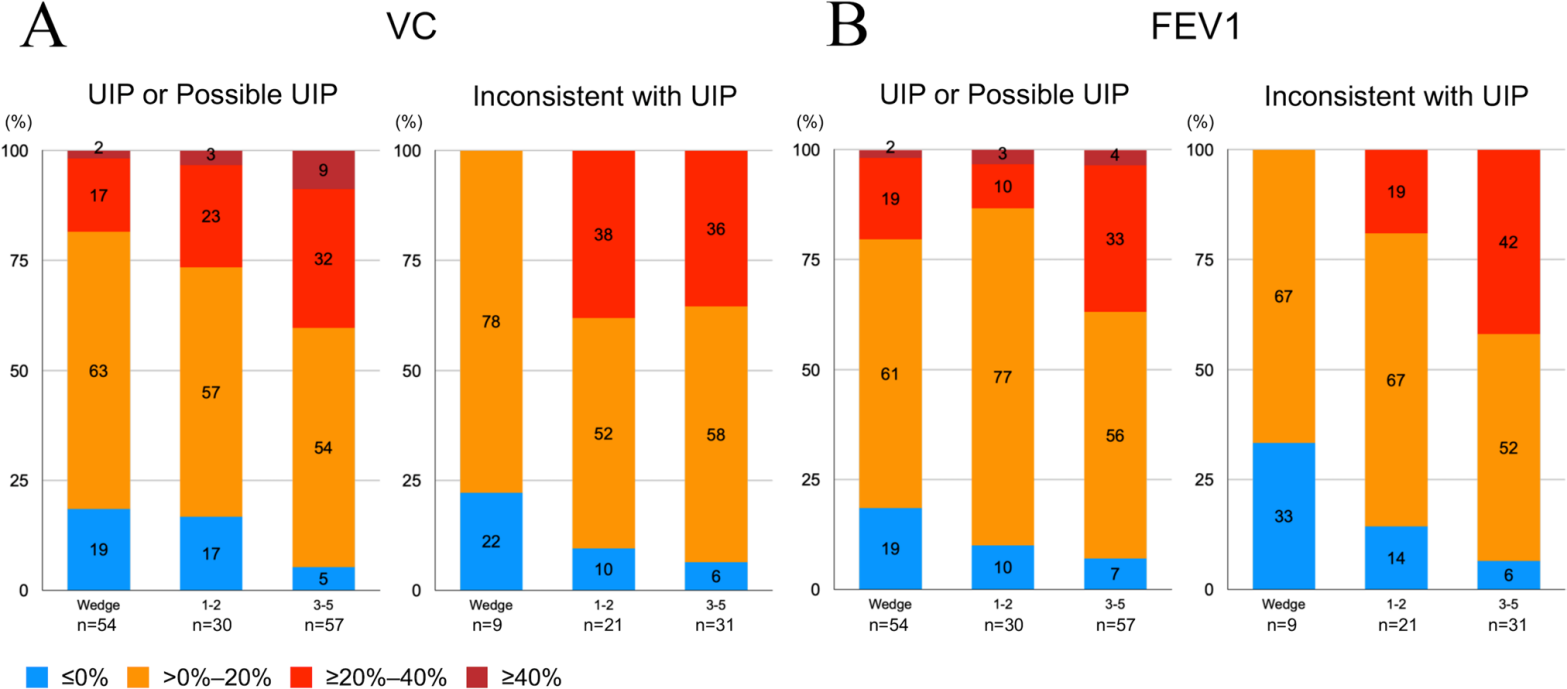
The percentage of patients according to the reduction rate at 6 months after surgery based on 20% intervals for VC [**A**] and FEV1 [**B**] of the UIP pattern or possible UIP pattern groups and the inconsistent with UIP pattern group. FEV1, forced expiratory volume in 1 s; UIP, usual interstitial pneumonia; VC, vital capacity

The number of patients with a reduction rate of ≥ 20% in VC and FEV1 at 6 months postoperatively was similar between the UIP pattern or possible UIP pattern groups and inconsistent with UIP pattern group [Fig. 4]. However, approximately 10% of patients in the UIP pattern or possible UIP pattern groups exhibited a decrease in the VC and FEV1 of ≥ 40%; this was not observed in patients in the inconsistent with UIP pattern group.

### Risk factors for long-term decreases in the postoperative pulmonary function

A multivariable logistic analysis showed that the risk factors for decreases in the postoperative reduction rates of VC [Table 2] and FEV1 [Table 3] differed between patients with and without ILAs. For patients with ILA, the number of resected segments was the only independent risk factor for long-term decreases in VC [3–5 segment resection vs. wedge resection: odds ratio [OR] = 3.33, 95% confidence interval [CI] = 1.50–7.43, P = 0.003] and FEV1 [3–5 segment resection vs. wedge resection: OR = 2.56, 95% CI = 1.17–5.63, P = 0.018; 3–5 segment resection vs. 1–2 segment resection: OR = 2.92, 95% CI = 1.22–6.99, P = 0.016]. In contrast, in the non-ILA group, male sex, lower preoperative diffusion capacity of the lung for carbon monoxide, number of resected segments, and postoperative complications were independent risk factors for long-term decreases in VC and FEV1.

**Table 2:** Univariable and multivariable logistic analyses of VC reduction rates ≥ 20% at 6 months

	ILA [n = 202]					Non-ILA [n = 989]			
	Univariable		Multivariable		Univariable			Multivariable	
	OR [95%CI]	P value	OR [95%CI]	P value	OR [95%CI]		P value	OR [95%CI]	P value
Age*, years	1.54 [0.32–7.37]	.648			1.01 [0.99–1.03]		.396		
Male vs. female patients	1.24 [0.62–2.51]	.542			1.96 [1.39–2.77]		< .001	1.76 [1.16–2.67]	.008
Brinkman index*	1.00 [0.99–1.00]	.735			1.00 [1.00–1.00]		< .001		
UIP pattern or possible UIP pattern vs. inconsistent with UIP pattern	0.91 [0.47–1.74]	.767			–		–	–	–
Predicted VC ≤ 80% vs. > 80%	1.37 [0.59–3.19]	.455			2.13 [1.27–3.57]		.004		
FEV1/FVC ≤ 70% vs. > 70%	1.00 [0.49–2.07]	.989			0.97 [0.66–1.45]		.895		
DLco ≤ 60% vs. > 60%	1.29 [0.62–2.67]	.494			2.08 [1.28–3.39]		.003	2.23 [1.32–3.76]	.003
Number of resected segments									
1–2 vs. wedge resection	2.42 [0.99–5.95]	.053	2.42 [0.99–5.95]	.053	1.28 [0.75–2.16]		.366	0.99 [0.51–1.96]	.995
3–5 vs. wedge resection	3.33 [1.50–7.43]	.003	3.33 [1.50–7.43]	.003	2.66 [1.64–4.28]		< .001	2.43 [1.28–4.62]	.006
3–5 vs. 1–2	1.37 [0.66–2.86]	.391	1.37 [0.66–2.86]	.391	2.08 [1.42–3.06]		< .001	2.44 [1.56–3.81]	< .001
Postoperative complications Yes vs. no	1.60 [0.81–3.17]	.178			3.13 [2.12–4.65]		< .001	2.61 [1.63–4.18]	< .001
Postoperative respiratory complications Yes vs. no	1.72 [0.83–3.60]	.146			3.51 [2.28–5.40]		< .001		
Adjuvant therapy Yes vs. no	1.21 [0.49–3.00]	.679			1.14 [0.72–1.81]		.580		

^*^Continuous value. *CI* confidence interval, *DLco* predicted diffusing capacity for carbon monoxide, *FEV*1 forced expiratory volume in 1 s, *FVC* forced vital capacity, *ILA* interstitial lung abnormality, *OR* odds ratio, *UIP* unusual interstitial pneumonia, *VC* vital capacity

**Table 3:** Univariable and multivariable logistic analyses of FEV1 reduction rates ≥ 20% at 6 months

	ILA [n = 202]					Non-ILA [n = 989]			
	Univariable		Multivariable		Univariable			Multivariable	
	OR [95%CI]	P value	OR [95%CI]	P value	OR [95%CI]		P value	OR [95%CI]	P value
Age*, years	1.01 [0.97–1.06]	0.622			0.99 [0.98–1.02]		0.948		
Male vs. female patients	0.89 [0.43–1.82]	0.744			1.36 [0.99–1.86]		0.058		
Brinkman index*	0.99 [0.99–1.00]	0.150			1.00 [0.99–1.00]		0.224		
UIP pattern or possible UIP pattern vs. inconsistent with UIP pattern	1.15 [0.57–2.33]	0.695			–		–	–	–
Predicted VC ≤ 80% vs. > 80%	1.54 [0.65–3.66]	0.331			1.53 [0.91–2.59]		0.123		
FEV1/FVC ≤ 70% vs. > 70%	0.63 [0.28–1.41]	0.254			1.19 [0.83–1.71]		0.354		
DLco ≤ 60% vs. > 60%	0.54 [0.24–1.16]	0.108			2.17 [1.36–3.46]		0.001	2.46 [1.49–4.06]	< .001
Number of resected segments									
1–2 vs. wedge resection	0.88 [0.32–2.38]	0.801	0.88 [0.32–2.38]	.801	0.78 [0.47–1.29]		0.332	0.63 [0.34–1.17]	.147
3–5 vs. wedge resection	2.57 [1.17–5.63]	0.018	2.57 [1.17–5.63]	.018	2.53 [1.65–3.89]		< 0.001	2.18 [1.24–3.85]	.007
3–5 vs. 1–2	2.92 [1.22–6.99]	0.016	2.92 [1.22–6.99]	.016	3.24 [2.19–4.81]		< 0.001	3.45 [2.21–5.37]	< .001
Postoperative complications Yes vs. no	0.64 [0.28–1.42]	0.272			1.61 [1.07–2.41]		0.022	1.85 [1.15–2.98]	.012
Postoperative respiratory complications Yes vs. no	0.77 [0.33–1.82]	0.558			1.83 [1.17–2.86]		0.008		
Adjuvant therapy Yes vs. no	1.02 [0.38–2.64]	0.976			1.40 [0.91–2.14]		0.126		

^*^Continuous value. *CI* confidence interval, *DLco* predicted diffusing capacity for carbon monoxide, *FEV*1 forced expiratory volume in 1 s, *FVC* forced vital capacity, *ILA* interstitial lung abnormality, *OR* odds ratio, *UIP* unusual interstitial pneumonia, *VC* vital capacity

## Discussion

The present study demonstrated that wedge resection can preserve the postoperative pulmonary function of patients with ILA as well as that in those without ILA; however, anatomical resection significantly decreased the postoperative pulmonary function of patients with ILAs compared with that of those without ILAs. We also found no significant difference in the postoperative reduction rate of pulmonary function between the UIP pattern or possible UIP pattern groups and inconsistent with UIP pattern groups. Thus, surgical procedures, especially anatomical resection, negatively affects the postoperative pulmonary function of patients with ILAs, regardless of the interstitial pattern on CT images.

Similarly, we have previously reported that interstitial pneumonia on CT images is a risk factor for a worse postoperative %VC decrease than predicted %VC [12], perhaps because lung fibrosis, which is characterised by thickening, stiffening, and scarring, may impair the expansion of the residual lung and its adaptation to the dead space in the thorax. Moreover, previous studies have shown that postoperative expansion or growth of the residual ipsilateral or contralateral lung is associated with the recovery of postoperative pulmonary function or relief from dyspnea syndrome [13, 14]. This study showed that improvements in pulmonary function at 6–12 months after surgery were not as great in patients with ILAs than in those without ILAs. Thus, when patients with ILAs lose their pulmonary function, it is difficult to restore that function due to impaired residual lung expansion or growth. Because the number of resected segments was the only risk factor for a decrease in the postoperative pulmonary function of patients with ILAs, the extent of pulmonary function loss depended on the extent of lung resection. The larger the extent of lung resection, the larger the dead space and loss of the vascular bed, which are substantial in patients with ILAs.

Furthermore, surgical invasion may be involved in the progression of ILAs leading to a decrease in the postoperative pulmonary function. Anatomical resection could result in a more serious insult to the residual lung than wedge resection because anatomical resection requires a longer operative time, hilar vascular procedures, lymph node dissection, and longer perioperative management [e.g., one-lung ventilation, positive pressure ventilation, and high concentration of oxygen exposure]. These factors seem to cause greater stress on the endothelium, which may result in the progression of ILAs and a long-term postoperative decrease in pulmonary function. Thus, the difference in surgical invasion between wedge resection and anatomical resection significantly affects the long-term postoperative decrease in pulmonary function. In addition, antifibrotic drugs, such as nintedanib, pirfenidone, and preferential phosphodiesterase 4B inhibitors, have been developed to prevent the progression of fibrosis and decreases in pulmonary function [15–17]. Perioperative use of these drugs may effectively prevent ILA progression, acute exacerbation, and decreased pulmonary function; however, prospective trials are required.

The present study demonstrated that the interstitial pattern on CT images was not a risk factor for decreased postoperative pulmonary function of patients with ILAs. The inconsistent with UIP pattern negatively affected postoperative pulmonary function to the same extent as the UIP pattern or possible UIP pattern. Some patients with the inconsistent with UIP pattern demonstrated a histological UIP [18]. Moreover, a previous study also showed that 32% of patients with a possible UIP pattern or inconsistent with UIP pattern developed the UIP pattern over a period of months to years [19]. Therefore, even if patients exhibit the inconsistent with UIP pattern, then postoperative pulmonary function loss should be considered when performing surgical procedures.

Preserving the pulmonary function is crucial to improving the long-term prognosis of patients with ILAs because decreased pulmonary function is associated with an increased risk of death [2, 20]. This study showed that wedge resection preserved the postoperative pulmonary function of patients with ILAs as much as it did in those without ILAs. These data support prior findings that wedge resection reduces mortality due to respiratory failure compared with lobectomy [7]. Minimising the loss of postoperative pulmonary function by wedge resection may restore pulmonary and physical functions and subjective health. However, sublobar resection for early-stage NSCLC patients with ILAs remains controversial. A previous report showed that wedge resection increased the number of deaths due to lung cancer compared with lobectomy for early-stage NSCLC patients with ILAs [7]; however, our previous study showed that sublobar resection, including wedge resection or segmentectomy, was feasible and resulted in acceptable outcomes compared with those of lobectomy [8]. Both cancer control and preservation of pulmonary function are important to improving the outcomes of NSCLC patients with ILAs. This study suggests that if the tumor is technically and oncologically resectable by sublobar resection, then minimising the degree of lung resection is recommended to restore the long-term pulmonary function. Definitive conclusions should be drawn based on ongoing prospective randomised clinical trials, such as JCOG1708, which compares the results of sublobar resection with those of lobectomy of early-stage NSCLC patients with ILAs [21].

This study had several limitations. First, this was a retrospective study including only patients who underwent pulmonary function tests 6 months postoperatively; those who died within 6 months of surgery or could not visit the hospital 6 months after surgery due to severe respiratory failure were excluded. Moreover, we did not measure the diffusion capacity of the lung for carbon monoxide, respiratory symptoms, and health status of patients, which are important for assessing the long-term outcomes of patients with ILAs. Furthermore, the present study used the 2011 ATS/ERS/JRS/ALAT classifications for CT; however, the interstitial pattern was reclassified into four categories according to the 2018 guidelines [22]. Use of the new classifications could have resulted in different results. These limitations should be considered when interpreting the results.

In conclusion, the decrease in long-term pulmonary function depends on the surgical procedure, but not on the interstitial patterns observed on CT images of early-stage NSCLC patients with ILA. Therefore, if the tumor is technically and oncologically resectable, then minimizing the extent of lung resection is recommended.

## Supplementary Information

Below is the link to the electronic supplementary material.

Supplementary file1 (DOCX 16 KB)

Supplementary file2 (DOCX 17 KB)

## Data Availability

The data that support the findings of this study are available from the corresponding author upon reasonable request.
