# Supplementary material for: Postoperative pulmonary function of patients with lung cancer and interstitial lung abnormalities
Source: Gen Thorac Cardiovasc Surg. 2024 May 9;72(12):786–95. doi: 10.1007/s11748-024-02037-7 (PMC11538201; doi:10.1007/s11748-024-02037-7)
Supplement: Supplementary file 1 — Supplementary file1 (DOCX 16 KB) [file 11748_2024_2037_MOESM1_ESM.docx]

Table S1: Changes in the postoperative reduction rates of VC and FEV1 at 6 and 12 months of the ILA and non-ILA groups.

|  |  | Reduction rate in VC | | | Reduction rate in FEV1 | |
| --- | --- | --- | --- | --- | --- | --- |
| Surgical procedure |  | 6 months | 12months | 6 months | | 12months |
| Wedge resection | ILA (n=63) | -6.82  (-12.41, -2.34) | -7.08  (-13.87, -0.85) | -7.05  (-13.04, 0) | | -5.33  (-16.24, 0) |
|  | Non-ILA (n=226) | -5.00  (-12.74, -0.52) | -4.83  (-11.07, 0) | -7.14  (013.14, -1.27) | | -6.49  (-12.96, -1.53) |
|  | P.value | 0.242 | 0.108 | 0.767 | | 0.809 |
| 1-2 segment resection | ILA (n=51) | -12.5  (-24.19, -11.09) | -15.02  (-22.26, -5.20) | -13.36  (-18.21, -6.74) | | -12.80  (-19.37, -6.22) |
|  | Non-ILA (n=342) | -9.93  (-16.36, -3.85) | -7.58  (-14.04, -2.31) | -10.27  (-16.26, -3.44) | | -8.38  (-14.64, -2.87) |
|  | P.value | 0.013 | <0.001 | 0.139 | | 0.010 |
| 3-5 segment resection | ILA (n=88) | -17.42  (-28.86, -11.08) | -16.96  (-24.75, -10.08) | -17.36  (-24.67, -9.79) | | -17.51  (-23.96, -9.47) |
|  | Non-ILA (n=421) | -14.23  (-21.57, -7.57) | -11.96  (-19.35, -5.29) | -14.39  (-20.93, -8.58) | | -14.30  (-20.60, -6.75) |
|  | P. value | 0.002 | <0.001 | 0.046 | | 0.025 |
| Values are expressed as the median (95% confidential interval). FEV1, forced expiratory volume in 1 second; ILA, interstitial lung abnormality; VC, vital capacity. | | | | | | |
