# Supplementary material for: Postoperative pulmonary function of patients with lung cancer and interstitial lung abnormalities
Source: Gen Thorac Cardiovasc Surg. 2024 May 9;72(12):786–95. doi: 10.1007/s11748-024-02037-7 (PMC11538201; doi:10.1007/s11748-024-02037-7)
Supplement: Supplementary file 2 — Supplementary file2 (DOCX 17 KB) [file 11748_2024_2037_MOESM2_ESM.docx]

Table S2: Changes in the postoperative reduction rates of VC and FEV1 at 6 and 12 months of the UIP pattern or possible UIP pattern and the inconsistent with UIP pattern group.

|  |  | Reduction rate in VC | | | Reduction rate in FEV1 | |
| --- | --- | --- | --- | --- | --- | --- |
| Surgical procedure |  | 6 months | 12months | 6 months | | 12months |
| Wedge resection | UIP or possible UIP (n=54) | -8.13  (-14.03, -2.79) | -6.87  (-14.69, -0.71) | -6.95  (-13.03, 4.65) | | -7.41  (-14.72, 2.56) |
|  | Inconsistent with UIP (n=9) | -2.55  (-6.25, 0.15) | -8.13  (-13.17, -2.81) | -7.32  (-13.87, -0.42) | | -7.35  (-17.15, 0) |
|  | P. value | 0.028 | 0.820 | 0.421 | | 0.930 |
| 1-2 segment resection | UIP or possible UIP (n=30) | -13.32  (-24.20, -3.82) | -13.51  (-22.10, -3.96) | -13.71  (-17.88, -7.45) | | -12.99  (-18.19, -7.63) |
|  | Inconsistent with UIP (n=21) | -12.5  (-28.58, -6.98) | -15.97  (-22.26, -6.80) | -11.89  (-20.03, -5.54) | | -10.81  (-19.89, -1.11) |
|  | P. value | 0.528 | 0.423 | 0.894 | | 0.778 |
| 3-5 segment resection | UIP or possible UIP (n=57) | -18.98  (-28.86, -10.05) | -17.12  (-26.91, -9.94) | -17.17  (-24.67, -8.26) | | -17.28  (-23.93, -9.72) |
|  | Inconsistent with UIP (n=31) | -15.08  (-27.96, -11.24) | -14.72  (-21.74, -10.02) | -17.72  (-25.58, -13.03) | | -18.24  (-24.58, -9.24) |
|  | P. value | 0.600 | 0.481 | 0.363 | | 0.945 |
| Values are expressed as the median (95% confidential interval). FEV1, forced expiratory volume in 1 second; UIP, usual interstitial pneumonia; VC, vital capacity. | | | | | | |
